# Editorial Notes

**Published:** 1914-09

**Authors:** 


### Editorial Notes.

**Annual Meeting of the B.M.A. at Aberdeen.**

THE British Medical Association Annual Meeting at Aberdeen was accounted by all who took part in it as one of the most interesting and successful of these gatherings, a result attributable to the splendid spirit of hospitality which animated not only the members of the local branch of the B.M.A., but the University and civic authorities, and also private individuals. Every hour of every day was filled with interest for the visitors. The place itself, with its long and varied history, and its many associations with great names and historic events in the past, was an admirable setting to the scientific business and social amenities of the occasion. Many must have wondered why the annual meeting had never before been held in such an eminently suitable place.

Sir Alexander Ogston's Presidential Address took place in the Music Hall, a building in the classical style which reminded Bristolians of the Victoria Rooms. There was a good attendance of members and ladies, and the proceedings began by the presentation of foreign and colonial visitors to the President. Unfortunately many of the former had already been recalled to their native countries owing to the disturbed political situation, and among the representatives of the British Dominions and Colonies there were also gaps, caused (it was surmised) by individual modesty, which shrank from the ordeal of mounting the platform. The address itself was a learned and interesting survey of the history of the Medical School in Aberdeen, which included also an account of the ethnology and political history of the district. The address supplemented well the several articles which have recently appeared in the *British Medical Journal* on the history and associations of the city and University, and was greatly appreciated by those who heard it.

The beautiful buildings of Marischal College, where the sectional and general meetings were held, must have impressed all visitors with their grace and dignity. Luckily several days were sunny, so that the sparkling grey granite of which they are built showed to great advantage. Opposite to these several of the visitors were shown by their hosts Aberdeen's only "slum." It would not be classed under that name in other towns we could mention, and consists apparently of a single narrow court where stands a tall granite house now let out in tenements. Formerly it was the abode of a Scottish lady who entertained the Duke of Cumberland there when he was quartered in Aberdeen. It is said that after his departure the family plate was found to have disappeared.

Many excursions and visits to places of interest were arranged and successfully carried out. On the afternoon of July 30th a well-filled special train took a number of visitors to Nordrach-on-Dee, near Banchory, where they were most hospitably entertained by Dr. and Mrs. Lawson. The sanatorium consists of one long building, the corridor and sanitary blocks being placed at the back facing the north, and the patients' rooms on the south, overlooking the Valley of the Dee. It is constructed of wood and iron, on a granite base, only the small central tower being of stone. At the back is a pretty lawn, surrounded with pine woods, where tea was provided. Patients said that it was nearly always possible to have tea out of doors. The rooms, which seemed very sunny and cheerful, are warmed by pipes running round the skirting. A fixed washing basin, with hot and cold water laid on, is provided in each. The windows are hinged, with lights opening horizontally above. At one end of the building are large iron verandahs, and at the other a " winter garden " opens on the south aspect. The pathological laboratory and the Medical Superintendent's house are in a separate building. Much interest was taken by visitors in the " kennels," where were seen fascinating white Highland terriers, Aberdeens and obsequious bull-dogs of extraordinarily aristocratic lineage. Another attraction was an open-air shooting gallery, where a distinguished London physician won a silver cigarette case for his accurate diagnosis of the bull's-eye. The dinner of the Association was excellent, and, what is more remarkable, the speeches afterwards were worthy of the dinner. The haggis was escorted in by pipers of the Gordon Highlanders, and tasted by most—especially as it was necessarily accompanied by some very superior neat whisky. Our northern colleagues assured us that this was physiologically the only sound practice.

Another interesting visit was that arranged for by Dr. E. I. Spriggs to Duff House. The party were taken in motor cars across fine open country for forty-five miles, which brought them to the north coast of Banffshire, and incidentally gave them a good appetite for lunch. After this function the party were shown over Duff House itself, and alternately admired the accuracy and elegance of diet sheets, and the beautiful decorations by William Adam, father of the two celebrated brothers. The mantelpieces and ceilings are as fine as anything which the architect's sons ever produced, and are shown to great advantage by the colouring, which has been recently renewed.

The diet sheets are skilfully drawn up, and show at a glance the caloric value and composition of all the food consumed by the patient ; the sugar and acetone bodies and other urinary constituents are also charted daily when necessary.

The River Deveron, which flows northward through the grounds, is of great natural beauty. The visitors were taken to a bridge, a mile or so above the house, where a single arch spans a rocky gorge lying deeply between wooded hills. Many felt that they would cheerfully face the dietetic regimen of Duff House for a month, tempered by frequent visits to the brown pools of the Deveron, and a reasonable amount of fisherman's luck. After tea the party motored back to Aberdeen, and the evening was devoted to the conversazione given by the Aberdeen Branch. It was held in the Music Hall, and consisted of a dance, a soirée, and a sort of smoking concert without the tiresome songs, all rolled into one. It formed a fitting conclusion to a very enjoyable gathering.

An excellent illustrated handbook, full of detail and admirably got up, was presented to the members by the local executive committee. By a strange irony we noticed among the advertisements one of that mysterious drug Umckaloabo, the origin of which has recently been discussed with considerable publicity.

**The War.**

THE sudden outbreak of a European war, whilst it disturbs profoundly the normal conditions of life in every civilised nation whether directly participating in the struggle or not, makes its effect felt in very direct fashion upon the medical profession. The demand for medical units in the field has called to the theatre of war not only the greater part of the regular establishments of the naval and military medical services, but these have had to reinforce their *personnel* with large additions to their junior ranks. Most of the hospitals in the country have had to surrender the majority of their resident officers to take commissions in the Navy or Army Medical Services. The mobilisation of the Territorial Force has drawn many more of our profession from their practices to train with their various field units ; whilst the Base or General Hospitals have been the means of withdrawing from some portion of their civil duties the honorary staffs of our leading hospitals. In Bristol the number of medical men serving with His Majesty's Forces in one capacity or another is very large. From the Royal Infirmary and General Hospital almost the whole of the resident staffs volunteered their services, and were at once accepted by the War Office. The vacancies thus created could not be filled by qualified men, and the senior students have had the opportunity of occupying the posts of house-physicians and house-surgeons, thus enjoying advantages and shouldering responsibilities which in times of peace are only accomplished after the qualifying examinations have been passed. It is no small credit to these men that they are proving themselves fully equal to the duties thrust prematurely upon them, and are discharging their responsibilities in such fashion that the normal work of the hospitals can continue without interruption or the slightest decline in efficiency. From our fleet and our army in the field the reports are too meagre to enable us to give any particular news of the Bristol Medicals who are on active service. Many of them are, we know, with the British Expeditionary Force, over whose movements a wise, if sometimes tantalising, obscurity prevails. We hope, before many months have passed, to welcome them back rich with the honours of a successful campaign, and to hear more of the services they are now rendering to the Allied Armies.

The Third South Midland Field Ambulance, under the command of Lieut.-Colonel James Young, T.D., are undergoing arduous training in the Eastern Counties preparatory to going out to take their place at the front.

Lieut.-Colonel J. Paul Bush, C.M.G., M.S., relinquishing all his other professional engagements for the time being to devote his whole time unreservedly to his arduous duties as Colonel-Commandant, has taken over the new Royal Infirmary (King Edward VII Memorial), and has there established the Second Southern General Hospital as half a general hospital, consisting of 260 beds for patients, and so far 50 per cent. of his *à la suite* staff of officers have been called up. Colonel Bush did not feel justified in insisting on the original scheme of the old and new buildings being taken over entirely by the military, in view of the fact that other adequate means for completing the scheme for 1,040 patients' beds had been placed at his disposal.

Colonel Bush has received most able assistance from his Adjutant, Captain F. C. Nichols, who is also devoting the whole of his time to the unit.

The scheme now stands as follows :—That provision is being made for what is equivalent to two general hospitals, each with 520 patients' beds, and which involves the further provision of 400 additional beds for the members of the medical staff, matrons, sisters, orderlies, and other supernumeraries being housed and fed, etc., in connection with such an establishment.

This increased accommodation has been obtained by the military authorities accepting the magnificent offer of the Bristol Board of Guardians to place at Colonel Bush's disposal their entire establishment at Southmead, where 680 patients may be received, if unfortunately the war should render necessary such a vast increase in the medical arrangements here. Furthermore, Mr. R. E. Bush has most generously placed at the service of the authorities 100 patients' beds, which he is equipping at his beautiful residence, Bishop's Knoll, Stoke Bishop, and which he has undertaken to maintain free of all expense, besides providing also the necessary additional accommodation and maintenance for the *personnel.*

We now enjoy the unfamiliar sight of various members of the Hospital and Infirmary staffs working side by side and wearing the R.A.M.C. uniform whilst on duty. The rank and file of the military hospital are installed as nursing orderlies acting under the direction of many of our sisters and nurses who belong to the Territorial Nursing Staff. The difficulties of organising and installing this military hospital have been ably overcome by Col. Bush and his staff, with the valuable assistance of Miss Baillie, Principal Matron, and Miss Harvey, Matron of the 2nd S. Gen. Hospital. The action of the President and Committee of the Bristol Royal Infirmary in placing, some years ago, the Infirmary buildings at the disposal of the War Office for this purpose has contributed to the prompt establishment of a singularly well-equipped military hospital, of which the authorities have not been slow to avail themselves. The first patients were admitted as early as August 13th, but it was not till September 2nd that the large contingent were received of the sick and wounded who had been continuously travelling from the fierce action at Mons. From all accounts the patients appreciate very highly the luxurious comforts by which they find themselves surrounded, and the stories they have to tell show that the British Army has lost none of its stoutness and gallantry. A striking feature of the nature of the injuries was the large number of men admitted suffering from wounds of the feet and legs inflicted by shrapnel bursting over them in the trenches. The German rifle fire seems to have been remarkably ineffective, and our men have a poor opinion of the marksmanship of their infantry.

The presence of our wounded soldiers in Bristol brings home to us in a way that we could not otherwise realise the sternness and magnitude of the struggle in which we are engaged against " Prussian militarismus," as a German friend rightly described it. At the same time, we believe it has already had the inestimable effect of impelling many men of serviceable age to join the colours and fill the lamentable gaps in our fighting force. We trust that a steady influx of recruits will enable our army in France and Belgium, no less than our fleet patiently waiting off Heligoland, " to entertain those that shall assail us with their own beef in their bellies, and before they eat of our Kentish capons," which Sir Walter Raleigh took to be the wisest way.

We append the names of the R.A.M.C. staff of the Second Southern General Hospital, the asterisk indicating those who have been mobilised up to the present.

Lieut.-Colonel J. PAUL BUSH, C.M.G., *Administrator.*
Captain F. C. NICHOLS, *Registrar.*
Lieut. A. L. TAYLOR, *Quartermaster.*

*Lieut.-Colonels.*

R. SHINGLETON SMITH.

*J. MICHELL CLARKE.

*G. MUNRO SMITH.

*Majors.*

*A. B. PROWSE.
*J. SWAIN.
C. A. MORTON.
P. WATSON-WILLIAMS.
*R. G. P. LANSDOWN.
C. H. WALKER.
W. R. ACKLAND.

*Captains.*

*F. H. EDGEWORTH.
*J. L. FIRTH.
*J. O. SYMES.
*T. CARWARDINE.
*JAS. TAYLOR.
H. G. KYLE.
*CAREY F. COOMBS.
*H. F. MOLE.
E. C. WILLIAMS.
*E. W. H. GROVES.
*J. M. FORTESCUE-BRICKDALE.
*E. H. E. STACK.
J. FREEMAN.
J. DACRE.
J. J. S. LUCAS.
H. E. HARRIS.
W. COTTON.
*HEDLEY HILL.
*W. S. V. STOCK.

*Honorary Medical Officers of the Bishop's Knoll Hospital.*

| | |
|---|---|
| Dr. J. A. Nixon, *Captain National Reserve.* | Mr. H. F. Parsons. |
| Mr. G. T. Myles. | Mr. A. Rendle Short. |

We have dealt only with the more purely medical and surgical provision in Bristol for the sick and wounded from the front, but we do not forget the tribute that is owing to those whose devotion and enterprise are so signally successful and deserving of grateful recognition in Red Cross work and the Ambulance, visiting the patients and providing for refugees and in numberless other directions. These we shall refer to in a subsequent issue.

**Winsley Sanatorium.**

In our June issue we drew attention to various grave defects in the management of the medical affairs of this institution, and incidentally alluded to their own President's criticism of the managing board, and to the resignation of their late resident medical officer, who found that he was not allowed sufficient administrative control, and that the number of patients and the inadequacy of trained assistants rendered impossible that individual care and attention which is the essence of modern sanatorium treatment. We further pointed out that before the end of last June one resident medical officer had had "*something like* one hundred patients to supervise and to treat" at a time.

At the ensuing meeting of the Bristol Health Committee some members, who are also associated with the Winsley Sanatorium, made themselves sponsors for that institution, though avoiding any reference to our first and indeed to most of the serious comments, and we much regret that they have made it necessary to return to the subject again.

Alderman Anstey said there were one or two of our statements that he felt it was his duty to refer to "as one who knew something about Winsley," and went on to state that "the daily average number of patients for 1913 was 73·7 . . . they had never yet reached 100." Well, 73 is bad enough if a maximum, yet even at that distant date the maximum number of patients had reached 85! But as the Alderman professed to be correcting our mistakes, it is a pity that it did not occur to him to mention that on the day he spoke there were no less than 96 patients at Winsley (which we still venture to call "something like" 100, and since that date they have reached 98), though this would have somewhat discounted the chafings of his colleague, Mr. Dyer, at our well-meant criticisms, put forward in the interests of poor patients suffering from a mortal disease.

Why, even if one most competent medical officer was granted the necessary and proper administrative power, he could not adequately carry out the work of controlling, treating, and supervising so many patients on anything like a high standard, and meanwhile regrettable occurrences at Winsley have shown that our strictures were not groundless. At Midhurst (King Edward VII's Sanatorium), with 104 beds, there are three medical officers and a resident pathologist, in addition to the consultants; here sound scientific work, which is of great value, is being done.

The medical men to whose initiative Winsley Sanatorium owes its existence, and who handed over the management to the Committee and Board of Management, were jealous for the good name of Winsley, and hoped that it would be continued at the higher level of efficiency as obtained when Mrs. Proctor Baker gave £10,000 to help especially the medical establishment. As matters are, Winsley is a warning to those who would allow the voluntary hospitals to drift into the control of a municipality. We gladly recognise the devotion of its managers—it is their discretion in medical affairs which is at fault; but since the Committee have lately been strengthened by a recognised expert on tuberculosis, one of the earlier resident medical officers of the Institution, and they are at length proposing to appoint a second medical officer, which their Medical Board urged long ago, we feel that the seed has not altogether fallen on stony ground. The War will make this tardy resolution less easy of accomplishment now, but we trust the remuneration offered will be on an adequate scale to attract the best skill for such highly responsible work.

---

